# Simultaneous Pancreas–Kidney Versus Kidney Transplant Alone: Real-World Outcomes in a Propensity-Matched Global Cohort

**DOI:** 10.3389/ti.2025.15709

**Published:** 2025-12-30

**Authors:** Davide Catarinella, Sarah Williford, Francesca Rusconi, Rossana Caldara, Lorenzo Piemonti

**Affiliations:** 1 Diabetes Research Institute, Istituto di Ricovero e Cura a Carattere Scientifico (IRCCS) Ospedale San Raffaele, Milan, Italy; 2 TriNetX LLC, Cambridge, MA, United States; 3 TriNetX Europe BV, Sint-Martens-Latem, Belgium; 4 Diabetes Research Institute, Universita Vita Salute San Raffaele, Milan, Italy

**Keywords:** simultaneous pancreas–kidney transplantation, kidney transplantation, diabetes mellitus, end-stage renal disease, patient survival

## Abstract

The true comparative effectiveness of simultaneous pancreas–kidney transplantation (SPKT) versus kidney transplantation alone (KTA) in patients with diabetes and end-stage renal disease remains incompletely defined. Using the TriNetX Global Collaborative Network (2010–2024), we identified 3,679 SPKT and 27,062 KTA recipients aged 18–59 years. In unmatched comparisons, SPKT recipients showed lower mortality, fewer cardiovascular events, and improved kidney graft survival relative to KTA recipients, but also higher early rejection, infection, and readmission rates. After 1:1 propensity score matching, the cohorts were well balanced across all measured covariates, and long-term estimates for survival (HR 1.00, 95% CI 0.90–1.10), kidney graft failure (HR 0.99, 95% CI 0.94–1.04), and cardiovascular events (HR 0.99, 95% CI 0.94–1.05) no longer differed over 10 years. In contrast, SPKT recipients maintained significantly lower HbA1c levels throughout follow-up (mean 6.2% vs. 6.6% at 5 years; p < 0.001), reflecting sustained physiologic glycaemic control and a high probability of insulin independence. Sensitivity analyses restricted to type 1 diabetes and non-obese recipients yielded consistent results. After accounting for measured differences between recipients, we did not detect a long-term survival advantage of SPKT over KTA, whereas durable metabolic benefits persisted. Because key donor and immunologic characteristics were not available, a modest intrinsic survival benefit cannot be excluded. These findings highlight the major role of patient selection and support individualised use of SPKT for metabolic indications and quality-of-life improvement rather than survival gain alone.

## Graphical Abstract

**Figure:**
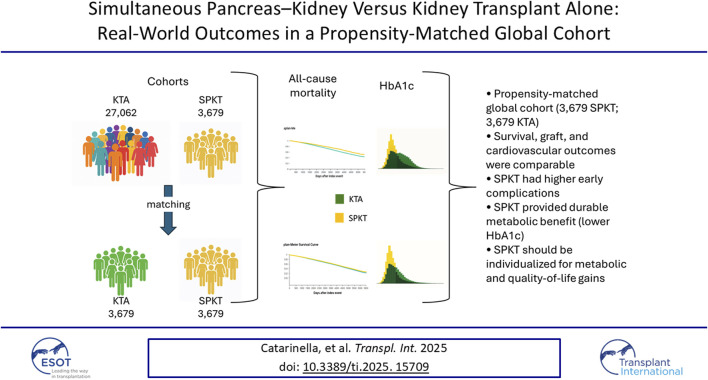

## Introduction

Simultaneous pancreas–kidney transplantation (SPKT) is a consolidated therapeutic option for patients with diabetes mellitus and end-stage renal disease (ESRD) who are eligible for pancreas transplantation [1–3]. By replacing both organs simultaneously, SPKT provides restoration of renal function together with endogenous insulin secretion, offering the potential for insulin independence and durable metabolic control [4–7]. Kidney transplantation alone (KTA) remains the most common approach worldwide due to its broader applicability, lower surgical complexity, and higher availability of organs, but it does not address the underlying diabetes or its long-term complications [8]. The theoretical advantages of SPKT extend beyond kidney graft survival and patient longevity [9]. Normalization of glycaemic control after successful pancreas transplantation improves HbA1c and reduces glycaemic variability, thereby decreasing the risk of acute metabolic decompensation and potentially preventing or slowing the progression of microvascular and macrovascular complications of diabetes [10–15]. Several observational studies have suggested that SPKT recipients achieve superior metabolic outcomes and quality of life compared with patients undergoing KTA, who remain insulin-dependent and often face suboptimal glucose control despite advances in medical therapy [16]. Despite these potential benefits, the impact of SPKT on hard clinical outcomes has been debated. Some registry-based analyses and single-centre reports have described lower mortality and cardiovascular events among SPKT recipients [17–22], particularly in type 1 diabetes [23, 24], while others have failed to confirm a survival advantage once differences in baseline risk profiles are accounted for [25–30]. Moreover, SPKT carries higher perioperative morbidity, increased immunosuppression, and greater risk of early complications, raising concerns about the overall balance of risks and benefits [31–33]. In the recent era, with improvements in surgical techniques [34–36], perioperative care [37–40], immunosuppressive strategies [41–43], and diabetes management [44], it remains unclear whether the historical advantages of SPKT over KTA persist in real-world practice. Importantly, while survival and graft outcomes are critical endpoints, the ability of SPKT to provide superior long-term glycaemic control represents a distinctive and clinically meaningful outcome that may translate into downstream benefits for patients. Large-scale real-world data may help clarify these uncertainties. TriNetX, a federated network of healthcare organizations, aggregates longitudinal electronic health records and enables comparative effectiveness research across diverse populations with robust analytic tools, including propensity score methods to mitigate baseline imbalances [45]. The objective of this study was to compare long-term outcomes of SPKT versus KTA in patients with diabetes and ESRD using the TriNetX Global Collaborative Network. We evaluated survival, kidney and pancreas graft outcomes, cardiovascular events, diabetes-related acute and chronic complications, malignancies, and mental health, with a particular focus on whether the improved glycaemic control achieved by SPKT translates into clinical benefit in the new era of transplantation.

## Materials and Methods

### Data Source and Ethics

We performed a retrospective cohort study using the TriNetX Global Collaborative Network (2010–2024, access date 23 September 2025), which aggregates de-identified EHR data from >150 healthcare organizations worldwide. The network provides demographics, diagnoses, procedures, laboratory values, medications, and vitals. Data are de-identified per HIPAA and GDPR; institutional review board approval and informed consent were not required for analyses of de-identified data.

### Study Population

Adults aged 18–59 years with diabetes and end-stage renal disease who underwent either simultaneous pancreas–kidney transplantation (SPKT) or kidney transplantation alone (KTA) were identified by transplant procedure codes. Exclusions: paediatric (<18 years) or older adults (>59 years), living-donor or multi-organ transplants, and records lacking a valid index date. The unmatched cohorts comprised 3,679 SPKT and 27,062 KTA recipients.

### Exposure, Index Event and Follow-Up

The exposure was transplant type (SPKT vs. KTA). The index event was the date of transplantation. For survival analyses (Kaplan–Meier and Cox regression), outcomes were assessed from 90 days post-transplant. For fixed-timepoint estimates, 1-year outcomes were calculated including events from day 10 post-transplant, while 5- and 10-year outcomes were calculated including events from day 90 onwards.

### Outcomes

Primary outcomes were (i) all-cause mortality, (ii) kidney graft failure, and (iii) death-censored graft failure. Secondary outcomes included: major adverse kidney events (MAKE: dialysis dependence, eGFR <15 mL/min/1.73 m^2^, transplant complications, or graft failure), transplant-related complications (ICD-10 T86.x), cardiovascular events (composite and components: acute myocardial infarction, stroke, heart failure, cardiac arrest, revascularization), infections/sepsis, treated acute rejection, 1-year hospital readmission, metabolic complications (hypoglycaemia; ketoacidosis/hyperosmolarity), microvascular complications (new-onset neuropathy; retinopathy), mental health (post-transplant depression/anxiety), and oncologic outcomes (PTLD/other neoplasms). Laboratory endpoints were most recent HbA1c and eGFR.

Detailed definitions of all outcomes, including the exact ICD-10 and procedure code lists used to define exposures, comorbidities and endpoints (e.g., cardiovascular events, rejection, infection, neuropathy), are provided in the Supplementary Methods. These definitions were pre-specified before any outcome analyses.

### Statistical Analysis

Comparative analyses between cohorts were performed using risk difference, risk ratio, and odds ratio with 95% confidence intervals, as well as Kaplan–Meier curves with log-rank tests and Cox proportional hazards regression. Propensity score matching (1:1 nearest-neighbour with caliper 0.1) was applied to balance baseline demographic, clinical, and laboratory covariates. For all Cox models we assessed the proportional hazards assumption visually and using Schoenfeld residuals; no major violations were detected. Further details on cohort definitions, index event and time windows, analytic settings, outcome definitions (including ICD, CPT, and laboratory codes), and propensity score methodology are reported in the Supplementary Methods.

## Result

### Baseline Characteristics

A total of 3,679 SPKT and 27,062 KTA recipients were identified. Before matching, SPKT recipients were younger, more often type 1 diabetic, and carried fewer cardiovascular comorbidities, whereas KTA recipients were more frequently of Black or Hispanic ethnicity and more commonly had ischemic heart disease, heart failure, dyslipidaemia, and obesity (Supplementary Table S1). After 1:1 propensity score matching, well-balanced pairs were generated with excellent covariate balance (all SMD <0.1; Supplementary Table S1; Supplementary Figures S1–S2). Median follow-up was ∼6 years in both groups. At the most recent assessment, HbA1c values were lower in SPKT compared with KTA recipients, both before matching (6.23% ± 1.68% vs. 7.11% ± 1.77%; p < 0.0001) and after matching (6.23% ± 1.68% vs. 6.58% ± 1.78%; p < 0.0001), although the difference was attenuated after adjustment. A similar pattern was seen for kidney function: eGFR was higher among SPKT recipients before matching (48.5 ± 29.3 vs. 44.1 ± 28.8 mL/min/1.73 m^2^; p < 0.0001), with only a modest residual difference after matching (48.5 ± 29.3 vs. 46.7 ± 29.0 mL/min/1.73 m^2^; p = 0.013).

### Primary Outcomes

**FIGURE 1:**
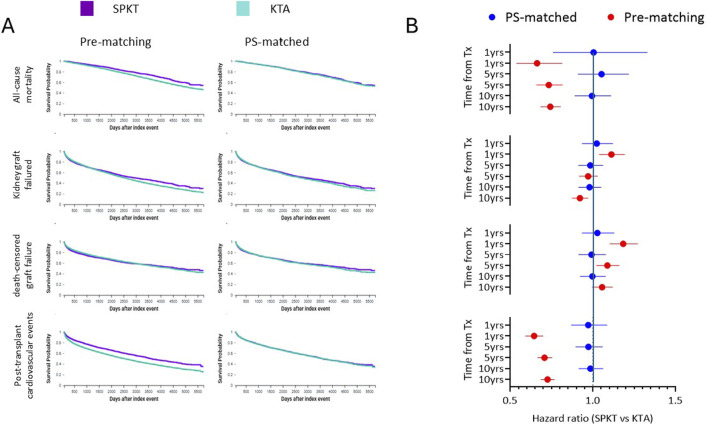
Kaplan–Meier survival curves and hazard ratios for SPKT versus KTA. **(A)** Kaplan–Meier estimates are shown for patient survival, overall graft survival, death-censored graft survival, and cardiovascular outcomes (major adverse cardiovascular events, myocardial infarction, stroke, and heart failure), comparing simultaneous pancreas–kidney transplantation (SPKT, purple) and kidney transplant alone (KTA, light blue). Curves are presented for unmatched cohorts (left column) and after 1:1 propensity score matching (right column). Follow-up extended up to 10 years. **(B)** The forest plot summarizes hazard ratios (HR, dots) with 95% confidence intervals (bars) for each outcome, calculated at prespecified timepoints (1, 5, and 10 years) in unmatched (red) and matched (blue) populations. HR values <1 indicate lower risk with SPKT, whereas HR values >1 indicate lower risk with KTA.

In the unmatched cohorts, SPKT recipients experienced significantly lower mortality compared with KTA, with hazard ratios well below unity and consistently favourable risk estimates at both 5 and 10 years (Table 1; Supplementary Tables S3–S4). Kaplan–Meier curves confirmed superior survival in SPKT (Figure 1). After propensity score matching, however, survival probabilities became virtually identical, and the risk of death did not differ between groups across all time points (Supplementary Tables S3–S4). Unadjusted Kaplan–Meier analysis suggested a modest advantage for SPKT, with lower cumulative incidence of graft loss over time (Table 1; Figure 1). However, risk estimates at 5 and 10 years indicated only minimal differences between groups, with relative risks close to unity (Supplementary Tables S3, S4). After propensity score matching, graft outcomes were fully comparable, with no evidence of a significant difference at any time point (Supplementary Tables S3, S4). In contrast, death-censored analyses showed less favourable outcomes for SPKT. In the unmatched population, the risk of death-censored graft failure was slightly higher in SPKT, particularly in the early post-transplant period, with relative risks favouring KTA (Supplementary Tables S3, S4). Kaplan–Meier curves showed largely overlapping trajectories (Figure 1). After matching, the differences disappeared, with similar risks of death-censored graft loss between groups (Table 1; Supplementary Tables S3, S4).

**TABLE 1:** Longitudinal outcomes (Kaplan–Meier and Cox models): SPKT vs. KTA.

Outcome	Cohort	Hazard ratio (95% CI)	KM log-rank p	Direction
All-cause mortality	PS-matched	1.00 (0.91–1.11)	0.97	Neutral
	Pre-matching	0.76 (0.71–0.83)	<0.001	Favors SPKT
Kidney graft failure	PS-matched	0.97 (0.91–1.04)	0.38	Neutral
	Pre-matching	0.92 (0.87–0.97)	0.001	Favors SPKT
Death-censored graft failure	PS-matched	0.99 (0.92–1.07)	0.79	Neutral
	Pre-matching	1.05 (0.99–1.11)	0.11	Neutral
MAKE	PS-matched	0.96 (0.91–1.01)	0.10	Neutral
	Pre-matching	0.82 (0.79–0.85)	<0.001	Favors SPKT
Post-transplant cardiovascular events	PS-matched	0.98 (0.91–1.05)	0.55	Neutral
	Pre-matching	0.73 (0.69–0.77)	<0.001	Favors SPKT
Treated acute rejection	PS-matched	1.02 (0.95–1.11)	0.57	Neutral
	Pre-matching	1.16 (1.09–1.23)	<0.001	Favors KTA
Acute myocardial infarction (first event)	PS-matched	1.09 (0.94–1.25)	0.26	Neutral
	Pre-matching	0.89 (0.80–0.99)	0.04	Favors SPKT
Heart failure (first event)	PS-matched	0.94 (0.84–1.05)	0.25	Neutral
	Pre-matching	0.70 (0.64–0.76)	<0.001	Favors SPKT
Stroke (first event)	PS-matched	1.05 (0.87–1.25)	0.63	Neutral
	Pre-matching	0.87 (0.76–1.00)	0.05	Favors SPKT
Infection or sepsis	PS-matched	1.00 (0.92–1.08)	0.98	Neutral
	Pre-matching	0.87 (0.82–0.93)	<0.001	Favors SPKT
Hypoglycaemia	PS-matched	1.00 (0.89–1.12)	0.93	Neutral
	Pre-matching	0.89 (0.81–0.97)	0.01	Favors SPKT
Ketoacidosis/hyperosmolarity	PS-matched	0.95 (0.83–1.09)	0.50	Neutral
	Pre-matching	1.20 (1.08–1.33)	0.001	Favors KTA
Depression/Anxiety onset post-Tx	PS-matched	0.99 (0.89–1.11)	0.87	Neutral
	Pre-matching	1.07 (0.98–1.16)	0.13	Neutral
Diabetic neuropathy (new onset)	PS-matched	1.11 (0.99–1.24)	0.06	Neutral
	Pre-matching	1.04 (0.96–1.14)	0.31	Neutral
Diabetic retinopathy (new onset)	PS-matched	1.06 (0.93–1.20)	0.38	Neutral
	Pre-matching	1.11 (1.00–1.22)	0.04	Favors KTA
PTLD/Neoplasm	PS-matched	1.01 (0.92–1.11)	0.87	Neutral
	Pre-matching	0.95 (0.88–1.01)	0.11	Neutral

Abbreviations. SPKT, simultaneous pancreas–kidney transplant; KTA, kidney transplant alone; KM, Kaplan–Meier; HR, hazard ratio; CI, confidence interval; PS-matched, propensity-score matched; MAKE, major adverse kidney events; PTLD, post-transplant lymphoproliferative disorder.

### Secondary Outcomes

A consistent pattern was observed across early peri-transplant endpoints. In the unmatched cohorts, SPKT recipients had higher rates of treated acute rejection, kidney transplant–related complications, and hospital readmission within the first year, all favouring KTA (Supplementary Tables S2–S4). After propensity score matching, the excess risk of acute rejection was no longer significant, whereas kidney transplant complications remained more frequent in SPKT, though with reduced effect sizes (Supplementary Tables S3-S4). Conversely, major adverse kidney events (MAKE) consistently favoured SPKT before adjustment, with hazard ratios and relative risks below unity across all time horizons (Table 1; Supplementary Tables S2–S4). After propensity score matching, however, this advantage was limited to the first post-transplant year, with neutral risks thereafter (Supplementary Table S2). In the unmatched cohorts, SPKT recipients showed lower risks of post-transplant cardiovascular events, with the advantage predominantly driven by a reduced incidence of heart failure (Table 1; Supplementary Tables S3–S4). Myocardial infarction and stroke occurred less frequently in SPKT as well, but the effect size was smaller. Kaplan–Meier analyses confirmed fewer cumulative cardiovascular events in SPKT, largely attributable to the divergence in heart failure risk (Figure 1). After propensity score matching, however, all differences were attenuated, and risks for the composite endpoint as well as for myocardial infarction, stroke, and heart failure became comparable between SPKT and KTA (Supplementary Tables S2–S4). In the unmatched cohorts, the profile of diabetes-related events was mixed. Diabetic ketoacidosis and hyperosmolar states were more frequent in SPKT, with relative risks favouring KTA (Supplementary Tables S3–S4). By contrast, severe hypoglycaemia occurred less often in SPKT, indicating a modest advantage for SPKT in this acute complication (Supplementary Tables S3–S4). For chronic complications, new-onset diabetic neuropathy and retinopathy were more frequent in SPKT, with risk estimates favouring KTA (Supplementary Tables S3–S4). After propensity score matching, however, all these differences were attenuated, and risks of acute decompensation, hypoglycaemia, neuropathy, and retinopathy became largely comparable between groups (Supplementary Tables S2–S4). Patterns of infection and sepsis varied according to the time horizon. In the unmatched cohorts, Kaplan–Meier estimates suggested slightly lower cumulative infection risk in SPKT over long-term follow-up (Supplementary Tables S3, S4). In contrast, early events within the first year were more common in SPKT, favouring KTA. After propensity score matching, the survival curves became largely overlapping, but the excess of early infections in SPKT persisted, while long-term risks converged toward neutrality (Supplementary Table S2). The incidence of post-transplant lymphoproliferative disease and other neoplasms was consistently similar between SPKT and KTA, both before and after adjustment (Supplementary Tables S3, S4). As a proxy of quality of life, new-onset depression or anxiety was slightly less frequent in KTA before matching, but this apparent difference was not confirmed after adjustment. In the matched cohorts, risks were virtually identical (Neutral; Supplementary Tables S2–S4).

### Sensitivity Analyses

To assess the robustness of our findings, we repeated all analyses in two restricted subgroups: (i) recipients with a primary diagnosis of type 1 diabetes (Supplementary Table S5), and (ii) type 1 diabetes recipients with a body mass index <30 kg/m^2^ at the time of transplantation (Supplementary Table S6). Across both sensitivity analyses, the direction and magnitude of risk estimates were consistent with those observed in the overall study population.

## Discussion

In this large, real-world analysis, SPKT recipients achieved consistently better glycaemic control than KTA recipients, as reflected by lower HbA1c levels both before and after propensity score matching. Despite this clear metabolic advantage, long-term patient survival, kidney graft survival, and cardiovascular outcomes were indistinguishable between SPKT and KTA once baseline differences were accounted for. The initial signals of improved survival and reduced cardiovascular risk in the unmatched cohorts were largely attributable to selection bias, with SPKT recipients being younger, predominantly affected by type 1 diabetes, and carrying fewer comorbidities at baseline. Importantly, SPKT was associated with higher early risks—including treated acute rejection, hospital readmission, perioperative complications, and infection/sepsis within the first post-transplant year. These excess short-term risks did not translate into inferior long-term outcomes. The only remaining clinical difference was a modest reduction in MAKE during the first post-transplant year, suggesting a possible short-term renoprotective effect of improved glycaemic control, although without sustained long-term impact on major endpoints. Our findings differ from the earliest registry-based and single-centre reports, which consistently suggested a survival and cardiovascular advantage of SPKT over KTA [46–49] particularly among younger recipients with type 1 diabetes [7, 21, 47, 49–51]. However, they align more closely with subsequent analyses that applied more comprehensive multivariable adjustment or propensity-based methods and reported attenuation or disappearance of these differences [25, 52, 53]. This pattern supports the interpretation that much of the apparent survival benefit of SPKT in historical cohorts may have reflected differences in recipient selection, donor quality, and the clinical context of earlier eras.

A notable result from our study is the persistently lower HbA1c observed in SPKT recipients after matching, despite the relatively small absolute difference (6.2% vs. 6.6%). Based on landmark trials such as DCCT/EDIC [54] and UKPDS [54], a 1% reduction in HbA1c corresponds to a 15%–20% reduction in microvascular risk and a 10%–15% reduction in cardiovascular events. Accordingly, the 0.3%–0.4% difference in our study would be expected to confer only a 4%–6% reduction in microvascular risk and a 3%–5% reduction in cardiovascular risk—an effect size insufficient to produce detectable long-term differences in survival or major cardiovascular outcomes in heterogeneous, real-world cohorts. This helps explain why improved glycaemic control after SPKT, while clinically relevant, did not translate into measurable survival advantages at a population level. These short-term risks associated with SPKT—including perioperative morbidity, treated rejection, infections, and early hospital readmissions—are well documented [31, 32, 36, 55, 56] and represent a recognised trade-off against the metabolic benefits. Furthermore, the therapeutic landscape has evolved substantially. Advances in continuous glucose monitoring, automated insulin delivery systems, and the availability of new agents such as SGLT2 inhibitors and GLP-1 receptor agonists have markedly improved glycaemic profiles and cardiovascular risk in patients with diabetes after kidney transplantation. These innovations have likely narrowed the incremental advantage of SPKT over KTA, further contextualising our findings of long-term similarity in hard outcomes.

This finding warrants further clinical interpretation. In SPKT recipients, an HbA1c in the low-to-mid 6% range reflects physiological insulin secretion, typically associated with minimal risk of severe hypoglycaemia and lower glycaemic variability. In contrast, similar HbA1c values in insulin-treated KTA recipients may mask substantial hypoglycaemia burden, glycaemic fluctuations, and the cognitive and emotional load of intensive insulin management. Because our dataset did not include continuous glucose monitoring metrics—such as time-in-range, glucose excursion indices, or asymptomatic hypoglycaemia—the true metabolic benefit of SPKT is likely underestimated. These considerations reinforce that the metabolic advantage of SPKT remains clinically meaningful even in the absence of detectable long-term survival differences. Our findings should also be interpreted in the context of prior evidence, which for decades has consistently shown a survival advantage of SPKT over KTA. Several factors likely explain why our real-world findings differ from these earlier observations. First, historical cohorts reflect an era of higher dialysis mortality and less effective diabetes and cardiovascular management. Second, donor and recipient selection practices have evolved: SPKT recipients typically receive younger, lower-risk organs and enter transplantation earlier in the course of diabetic complications, whereas KTA recipients accumulate greater comorbidity and longer pre-transplant dialysis exposure. These factors likely amplified earlier survival signals. Third, improvements in perioperative care, modern immunosuppression, and cardiovascular therapy have narrowed the survival gap. Finally, because our dataset lacked key transplant-specific variables—such as donor quality indices, HLA matching, cold ischaemia time, and immunosuppression—an intrinsic survival benefit of SPKT cannot be excluded and may be masked by unmeasured confounding. Together, these considerations reconcile our findings with the broader literature and suggest that, in current practice, the dominant advantage of SPKT lies in its metabolic and quality-of-life benefits rather than in large differences in long-term survival.

This study has several important limitations First, despite rigorous propensity score matching, residual confounding is unavoidable because the TriNetX platform lacks key transplant-specific variables. Donor quality metrics such as Kidney Donor Profile Index (KDPI) and Pancreas Donor Risk Index (PDRI), which strongly influence kidney outcomes and differ systematically between SPKT and KTA, were not available. Similarly, no information was provided on HLA matching, panel reactive antibodies, donor-specific antibodies, cold ischaemia time, centre experience or detailed immunosuppression protocols. These unmeasured factors may attenuate or obscure a true intrinsic survival benefit of SPKT or, conversely, magnify early procedural risk. Second, exposures, comorbidities and outcomes were identified using ICD-10 and procedure codes. The complete lists of codes used in this study are provided in the Supplementary Methods. Although these coding-based definitions follow established conventions, they remain prone to misclassification, under-reporting and variability across institutions—particularly for complex outcomes such as cardiovascular events, rejection, infection or neuropathy, for which clinical adjudication would be preferable. Third, the database does not capture patient-reported outcomes, continuous glucose monitoring metrics or hypoglycaemia burden—elements that represent the most meaningful clinical benefits of SPKT for many patients [57, 58]. As a result, the metabolic advantage observed in this study likely underestimates the full quality-of-life impact of successful pancreas transplantation [59, 60]. Fourth, diabetes type was defined using diagnosis codes, which may misclassify insulin-treated type 2 diabetes as type 1. Although sensitivity analyses restricted to patients coded as type 1 diabetes and to non-obese recipients were performed, some residual misclassification may persist. Finally, because SPKT by definition requires a deceased donor, our comparison group included only deceased-donor KTA recipients. These findings cannot be extrapolated to living-donor kidney transplantation, which often provides superior survival and represents a distinct clinical pathway.

Taken together, these limitations suggest that while our findings demonstrate no detectable long-term survival advantage of SPKT after adjustment for measured variables, a modest true benefit cannot be excluded. Rather, our results underscore the extent to which survival outcomes are shaped by patient selection, donor quality, and centre-level variation. In this context, the principal justification for SPKT in contemporary practice lies in its profound metabolic and quality-of-life benefits, balanced against higher early procedural risks.

In summary, this large, contemporary real-world analysis shows that the apparent survival advantage of SPKT over KTA disappears after balancing for measurable clinical covariates. Because donor quality and other key transplant-specific factors were not captured, a residual survival benefit cannot be excluded. Nevertheless, SPKT provides durable metabolic benefits, including excellent glycaemic control and freedom from insulin. In the setting of comparable observed survival, decisions about SPKT should be individualised, considering each patient’s preference for insulin independence, glycaemic stability, and quality-of-life improvement, as well as willingness to accept higher short-term risks. These findings also highlight a broader issue: despite clear metabolic and quality-of-life benefits, SPKT remains underutilised, and many eligible patients are not systematically referred to transplant centres. Variability in referral pathways, limited awareness among non-transplant clinicians, and the absence of structured evaluation frameworks likely prevent equitable access. In light of our results—showing that the decision for SPKT increasingly centres on metabolic benefit and patient preference—timely and systematic referral becomes critical. Strengthening referral pathways and enhancing collaboration between diabetologists, nephrologists, and transplant teams will be essential to ensure that all suitable candidates are appropriately evaluated.

## Data Availability

Individual participant data will not be made available. Study protocol, statistical analysis plan, and analytical code will be available from the time of publication in response to any reasonable request addressed to the corresponding author.

## Supplementary Material

SUPPLEMENTARY FIGURE S1 Standardized mean differences (SMD) for baseline characteristics between SPKT and KTA recipients before (red circles) and after propensity score matching (blue squares). Variables include demographics, comorbidities, and diabetes type. Dashed vertical lines at ±0.1 indicate the threshold for acceptable balance. Matching achieved excellent covariate balance across all variables, with all post-matching SMDs <0.1.

SUPPLEMENTARY FIGURE S2 Distribution of propensity scores, follow-up time, HbA1c, and eGFR before and after matching. The figure displays cumulative distribution curves of propensity scores (top panels), histograms of follow-up time (middle panels), and histograms of last available laboratory values for HbA1c (bottom left) and eGFR (bottom right) in SPKT and KTA recipients, shown separately for unmatched and propensity score–matched cohorts.
