# Supplementary figures and images for: Simultaneous Pancreas–Kidney Versus Kidney Transplant Alone: Real-World Outcomes in a Propensity-Matched Global Cohort
Source: Transpl Int. 2025 Dec 30;38:15709. doi: 10.3389/ti.2025.15709 (PMC12797426; doi:10.3389/ti.2025.15709)

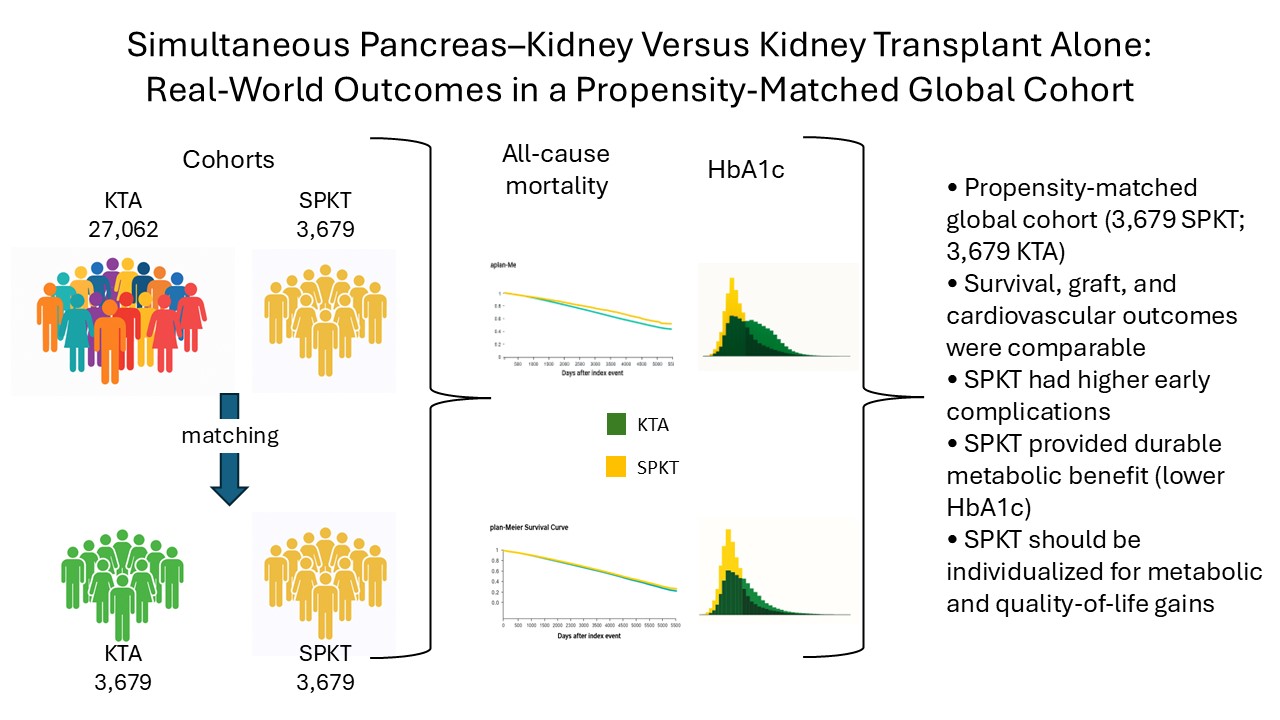

Supplement: Supplementary file 1 [file Image3.jpeg]

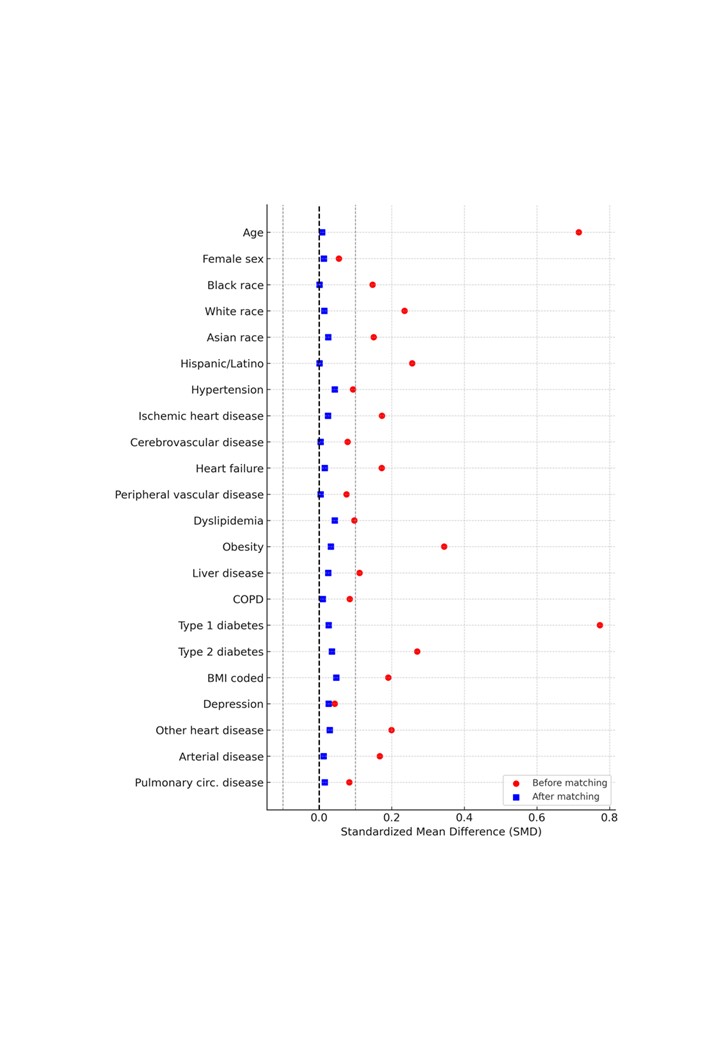

Supplement: Supplementary file 4 [file Image1.jpeg]

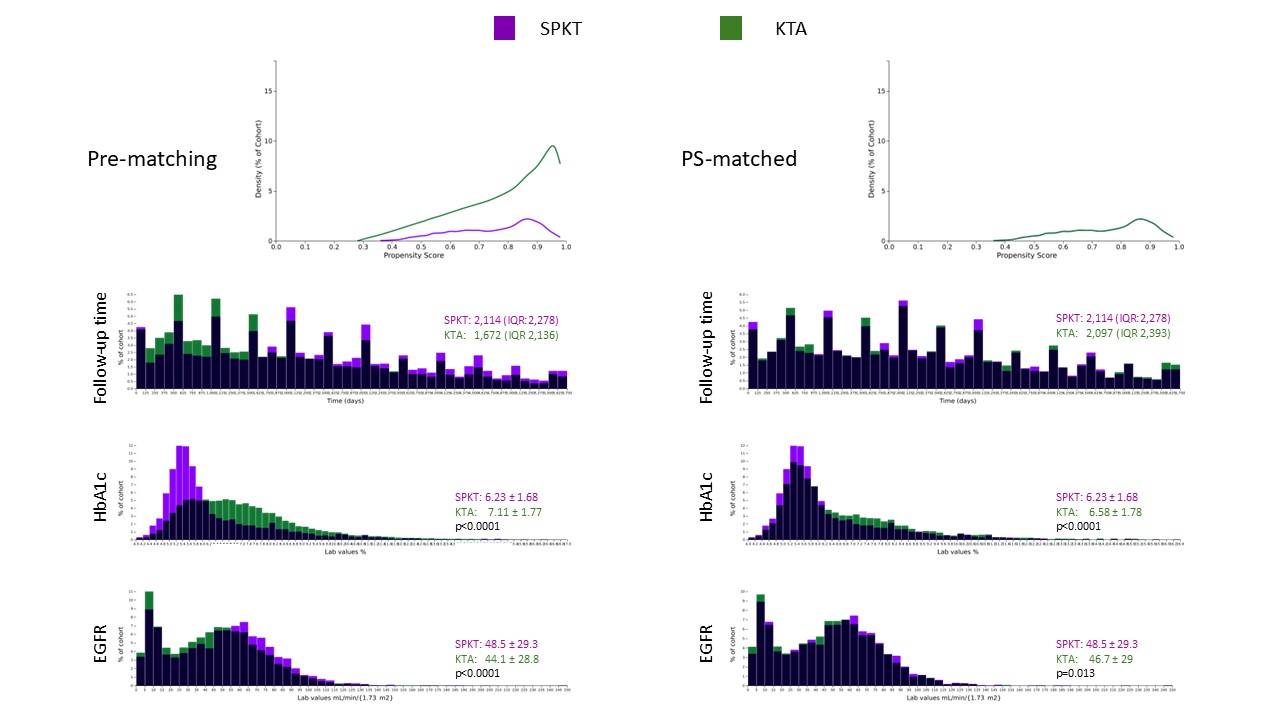

Supplement: Supplementary file 5 [file Image2.jpeg]
