# Supplementary material for: Simultaneous Pancreas–Kidney Versus Kidney Transplant Alone: Real-World Outcomes in a Propensity-Matched Global Cohort
Source: Transpl Int. 2025 Dec 30;38:15709. doi: 10.3389/ti.2025.15709 (PMC12797426; doi:10.3389/ti.2025.15709)
Supplement: Supplementary file 2 [file Supplementaryfile1.docx]

**Supplementary Methods**

**1. Data Source**

This study used the TriNetX Global Collaborative Network, a federated research platform aggregating de-identified electronic health records (EHRs) from >150 healthcare organizations worldwide. Available data include demographics, diagnoses (ICD-10-CM), procedures (ICD-10-PCS, CPT, HCPCS), medications (RxNorm, ATC), laboratory values (LOINC), and mortality. Data quality is ensured through systematic checks of conformance, completeness, and plausibility. All data are de-identified in compliance with HIPAA and GDPR; thus, studies are exempt from institutional review board approval.

**2. Study Population and Cohorts**

The study population included adult patients with diabetes mellitus and end-stage renal disease (ESRD) who underwent kidney transplantation between January 1, 2010 and December 31, 2024. Eligible recipients were aged 18 to 59 years at the time of transplant and had either a diagnosis of type 1 or type 2 diabetes (ICD-10-CM: E08–E13, E10, E11) together with documented ESRD or dialysis dependence (ICD-10-CM N18.6, Z99.2; CPT 90935, 90937, 1012740). Patients were excluded if they were younger than 18 or older than 59 years, if they received a living donor kidney transplant, or if they underwent multi-organ transplantation other than simultaneous pancreas–kidney transplantation (SPKT). Cases in which the index transplant event had occurred more than 20 years before data extraction were also excluded. Cohorts were then defined according to the presence or absence of a pancreas transplant. SPKT recipients were identified by the combination of kidney and pancreas transplantation within ±30 days, or by specific HCPCS codes, whereas kidney transplant alone (KTA) recipients were defined by the absence of any pancreas transplant procedure. Details of the coding algorithms are summarized in Table 1.

**Table 1. Cohort definitions and codes**

| Cohort | Definition | Codes |
| --- | --- | --- |
| SPKT (Simultaneous Pancreas–Kidney Transplant) | Diabetes + ESRD/dialysis + kidney + pancreas transplant within ±30 days, or HCPCS SPKT code | Diabetes: ICD-10-CM E08–E13, E10, E11  ESRD/dialysis: ICD-10-CM N18.6, Z99.2; CPT 90935, 90937, 1012740  Kidney Tx: ICD-10-CM Z94.0; CPT 50360, 50365; ICD-10-PCS 0TY00Z0, 0TY10Z0  Pancreas Tx: ICD-10-CM Z94.83; CPT 48554; ICD-10-PCS 0FYG0Z0  Simultaneous Tx: HCPCS S2065 |
| KTA (Kidney Transplant Alone) | Diabetes + ESRD/dialysis + kidney transplant with no pancreas transplant | Same diabetes/ESRD/dialysis/kidney codes as above; no pancreas codes |
| Exclusions | Pediatric/older age, multi-organ, living donor | Heart Tx: ICD-10-CM Z94.1  Lung Tx: Z94.2  Liver Tx: Z94.4 |

**3. Index Event and Time Windows**

The index event for all analyses was defined as the date of kidney transplantation, with or without a simultaneous pancreas graft. To ensure consistency across endpoints, different observation windows were applied. For survival analyses, including Kaplan–Meier estimates and Cox proportional hazards models, follow-up began 90 days after the transplant in order to exclude the perioperative period. For fixed-timepoint outcomes, events within the first year were assessed starting from day 10 post-transplant, while outcomes at 5 and 10 years were evaluated beginning from day 90 onward. Patients were followed until the occurrence of the event of interest or censored at the time of their last available record, with a maximum follow-up of 20 years.

**4. Outcomes**

The study evaluated a comprehensive set of post-transplant outcomes, defined using standardized diagnostic, procedural, and laboratory codes within TriNetX. The **primary outcomes** were all-cause mortality, kidney graft failure (considered both as a composite including death and as death-censored).

**Secondary outcomes** encompassed several domains. Kidney-related outcomes included major adverse kidney events (MAKE), defined as dialysis dependence, severely reduced renal function (eGFR ≤15 mL/min/1.73m²), graft complications, or failure. Transplant-related complications were captured through specific codes for kidney graft complications, while immunological outcomes included treated acute rejection. Cardiovascular events comprised acute myocardial infarction, stroke, heart failure, cardiac arrest, and revascularization procedures. Further secondary outcomes addressed infectious complications (sepsis or transplant-related infections), hospital readmission within one year, and metabolic or long-term complications. These included most recent laboratory values (HbA1c and eGFR), hypoglycemia, diabetic ketoacidosis or hyperosmolar states, as well as microvascular complications such as new-onset diabetic neuropathy and diabetic retinopathy. Mental health diagnoses (depression or anxiety after transplantation) and oncologic outcomes (post-transplant lymphoproliferative disease [PTLD] or other malignancies) were also assessed. The full list of outcome definitions and coding strategies is reported in **Table 2**.

**Table 2. Outcome definitions and codes**

| Outcome | Definition | Codes |
| --- | --- | --- |
| All-cause mortality | Deceased status | ICD-10-CM R99; TriNetX “Deceased” flag |
| Kidney graft failure (composite) | Graft failure (return to dialysis, re-Tx, failure code) or death | ICD-10-CM T86.12; CPT 50360, 50365; dialysis: CPT 90935, 90937, 1012740; ICD-10-CM Z99.2; mortality R99 |
| Death-censored graft failure | Graft failure excluding death | Same as above without mortality |
| Complications of kidney Tx | Transplant complications | ICD-10-CM T86.1, T86.10, T86.19 |
| Major adverse kidney events (MAKE) | Dialysis dependence, eGFR <15, complications, or graft failure | Codes above + TNX:8001 (eGFR ≤15) |
| Cardiovascular events | Acute MI, stroke, heart failure, cardiac arrest, revascularization | MI: I21; Stroke: I63.x; HF: I50; Arrest: I46 |
| Infection/sepsis | Sepsis or transplant infection | ICD-10-CM A41, B99, T86.13 |
| Treated acute rejection | Kidney rejection | ICD-10-CM T86.11 |
| Hospital readmission (1y) | Any inpatient encounter within 12m | HL7V3 inpatient encounter |
| Labs | Most recent lab values | HbA1c: TNX:9037; eGFR: TNX:8001 |
| Hypoglycemia | Hypoglycemia ± coma | E16.0, E16.1, E16.2, R56 |
| DKA/Hyperosmolarity | Ketoacidosis, hyperosmolar state | E10.1x, E11.0x |
| Diabetic neuropathy | Neuropathy, autonomic, amyotrophy, gastroparesis | E11.4, E11.43, E11.44, K31.84, G90.09 |
| Diabetic retinopathy | Non-proliferative, proliferative, ± macular edema | E11.3x, E11.32x, E11.33x, E11.34, E11.35, E11.36 |
| Mental health outcomes | Depression or anxiety post-Tx | F32.9, F41 |
| PTLD / Neoplasms | PTLD or other malignancies | C85.8, C85.9, C00–D49 |

**5. Statistical Analyses**

All analyses were conducted using the TriNetX “Compare Outcomes” analytics module. Categorical outcomes were summarized as absolute risks, with risk differences (RD), risk ratios (RR), and odds ratios (OR) reported together with 95% confidence intervals. For time-to-event outcomes, survival functions were estimated by the Kaplan–Meier method and compared between groups using the log-rank test. Multivariable Cox proportional hazards regression was additionally applied to estimate hazard ratios (HRs) with 95% confidence intervals. The proportional hazards assumption was verified using Schoenfeld residuals. Patients were censored at the time of their last available record or at a maximum of 20 years post-transplant, whichever occurred first. For laboratory outcomes, the most recent available value of HbA1c and estimated glomerular filtration rate (eGFR) within the follow-up window was extracted for each patient. Analyses were conducted both in the overall unmatched cohorts and after adjustment through propensity score matching (PSM). To account for baseline differences between groups, we applied 1:1 greedy nearest-neighbor matching with a caliper width of 0.1 of the pooled standard deviation of the logit of the propensity score. The propensity score was estimated using a logistic regression model that incorporated a broad range of clinically relevant covariates. These included demographic variables (age, sex, and race/ethnicity categories: Black/African American, White, Asian, Hispanic/Latino, and Other/Unknown), cardiovascular comorbidities (hypertension, ischemic heart disease, cerebrovascular disease, heart failure, peripheral vascular disease, other heart disease, arterial disease, and pulmonary circulation disease), and systemic or metabolic conditions (dyslipidemia, obesity, liver disease, and chronic obstructive pulmonary disease). Diabetes-related variables (type 1 and type 2 diabetes) and mental health status (history of depression) were also incorporated into the model. Covariate balance between SPKT and KTA recipients was assessed by standardized mean differences (SMDs). An SMD below 0.1 was prespecified as the threshold for adequate balance, and post-matching assessments confirmed that all covariates achieved this criterion, supporting the robustness of subsequent outcome comparisons.

**7. Flow of Patient Selection**

The derivation of the analytic cohorts followed a stepwise process within the TriNetX Global Network and is detailed in **Table 3**. Starting from the entire population of more than 170 million patients available, successive filters were applied to narrow the study sample. First, we identified all individuals with a diagnosis of diabetes mellitus, then restricted the population to those with end-stage renal disease (ESRD) or dialysis dependence. From this group, patients who had undergone kidney transplantation were selected. At this stage, the population was further stratified into those who received a simultaneous pancreas–kidney transplant (SPKT) and those who received a kidney transplant alone (KTA). Finally, standard inclusion and exclusion criteria were applied, limiting the analysis to adults aged 18–59 years, excluding living donor and multi-organ transplants, and restricting to index events within the last 20 years. As these criteria were already built into the initial TriNetX query, the numbers did not change after step 5. The final analytic cohorts consisted of **3,679 SPKT** and **27,062 KTA** recipients.

**Table 3. Cohort attrition and inclusion criteria**

| **Step** | **Description** | **N (patients)** |
| --- | --- | --- |
| 1 | All patients in TriNetX Global Network (2010–2024) | 170,455,599 |
| 2 | Patients with diabetes mellitus (ICD-10-CM E08–E13, E10, E11) | 11,554,862 |
| 3 | Diabetic patients with ESRD/dialysis (ICD-10-CM N18.6, Z99.2; CPT 90935, 90937, 1012740) | 521,648 |
| 4 | Diabetic patients undergoing kidney transplantation (ICD-10-CM Z94.0; CPT 50360, 50365) | 38,280 |
| 5 | Split into cohorts: SPKT (kidney + pancreas) vs. KTA (kidney alone) | SPKT = 3,679 / KTA = 27,062 |
| 6 | Apply inclusion/exclusion criteria: age 18–59, first transplant, exclude living donor, exclude multi-organ (heart, lung, liver), index event within 20 years | Final cohorts unchanged: SPKT = 3,679 / KTA = 27,062 |

**8. Technical Notes on Denominators**

The unmatched reference cohorts comprised **3,679 SPKT** and **27,062 KTA** recipients, representing all patients who satisfied the inclusion and exclusion criteria at the time of their index transplant event. However, the number of evaluable patients varied across outcome analyses, and this variability is explained by two key factors. First, **outcome-specific exclusions** were automatically applied by TriNetX. Patients with a prevalent diagnosis of the outcome of interest before the start of the observation window, insufficient follow-up, or missing laboratory data were excluded from the relevant analysis. This procedure ensured appropriate comparability but resulted in slight differences in denominators across endpoints in the unmatched cohorts. Second, **propensity score matching (PSM)** influenced the analytic sample sizes. The PSM algorithm only includes patients with complete covariate data available for balancing. As a result, the denominators in the matched analyses could be either higher or lower than those observed in the unmatched analyses. This occurs because patients excluded initially due to missing covariate data may be reintroduced into the analytic set once suitable matches are identified. Taken together, these processes account for the variability in denominators across outcomes, while ensuring that each analysis was conducted on the most valid and balanced population available.

**9. Handling of missing data**Propensity score models included only baseline demographic and comorbidity variables with >95% completeness at the index date; laboratory variables (e.g. HbA1c, eGFR) were not used for matching. Propensity scores and matching were therefore based on a complete-case analysis for those covariates. For longitudinal laboratory outcomes, we analysed all participants with at least one value available in the specified time window and did not perform imputation.
