# Supplementary material for: Simultaneous Pancreas–Kidney Versus Kidney Transplant Alone: Real-World Outcomes in a Propensity-Matched Global Cohort
Source: Transpl Int. 2025 Dec 30;38:15709. doi: 10.3389/ti.2025.15709 (PMC12797426; doi:10.3389/ti.2025.15709)
Supplement: Supplementary file 3 [file Table1.docx]

**Supplementary tables**

**Table 1. Baseline characteristics of SPKT and KTA recipients before and after propensity score matching**

| **Variable** | **Pre-matching** | | | **PS-Matched** | | |
| --- | --- | --- | --- | --- | --- | --- |
|  | **SPKT** | **KTA** | **SMD** | **SPKT** | **KTA** | **SMD** |
| Age, years (mean ± SD) | 44.1 ± 8.6 | 51.2 ± 11.1 | 0.715 | 44.1 ± 8.6 | 44.0 ± 9.2 | 0.008 |
| Female sex, % | 40.3 | 37.6 | 0.054 | 40.3 | 39.7 | 0.013 |
| Male sex, % | 58.1 | 61.1 | 0.061 | 58.1 | 59.0 | 0.017 |
| Black/African American, % | 23.7 | 30.2 | 0.147 | 23.7 | 23.7 | 0.001 |
| White, % | 58.0 | 46.3 | 0.235 | 58.0 | 57.3 | 0.014 |
| Asian, % | 2.8 | 5.8 | 0.150 | 2.8 | 3.2 | 0.025 |
| Hispanic/Latino, % | 7.1 | 15.1 | 0.256 | 7.1 | 7.1 | 0.001 |
| Other/Unknown race, % | 14.9 | 17.5 | ~0.10 | 14.9 | 14.5 | <0.03 |
| Hypertension, % | 58.8 | 63.3 | 0.093 | 58.8 | 56.6 | 0.043 |
| Ischemic heart disease, % | 16.6 | 23.5 | 0.173 | 16.6 | 15.7 | 0.024 |
| Cerebrovascular disease, % | 4.1 | 5.8 | 0.078 | 4.1 | 4.0 | 0.004 |
| Heart failure, % | 6.7 | 11.6 | 0.172 | 6.7 | 6.3 | 0.015 |
| Peripheral vascular disease, % | 4.7 | 6.5 | 0.075 | 4.7 | 4.8 | 0.004 |
| Dyslipidaemia, % | 31.7 | 36.3 | 0.097 | 31.7 | 29.7 | 0.043 |
| Obesity, % | 4.9 | 15.1 | 0.344 | 4.9 | 4.2 | 0.032 |
| Liver disease, % | 2.5 | 4.5 | 0.111 | 2.5 | 2.1 | 0.025 |
| COPD, % | 1.1 | 2.1 | 0.084 | 1.1 | 1.0 | 0.010 |
| Type 1 diabetes, % | 52.8 | 18.3 | 0.773 | 52.8 | 51.4 | 0.026 |
| Type 2 diabetes, % | 46.1 | 59.5 | 0.270 | 46.1 | 44.4 | 0.035 |
| Depression (history), % | 8.0 | 6.9 | 0.043 | 8.0 | 7.3 | 0.026 |
| Other heart disease, % | 20.5 | 29.1 | 0.199 | 20.5 | 19.4 | 0.029 |
| Arterial disease, % | 10.6 | 16.3 | 0.167 | 10.6 | 10.3 | 0.012 |
| Pulmonary circulation disease, % | 3.5 | 5.2 | 0.083 | 3.5 | 3.2 | 0.015 |

**Supplementary Table 2. One-year absolute and relative risks (SPKT vs KTA)**

| **Outcome** | **Cohort** | **AR**  **SPKT (1y)** | **AR**  **KTA (1y)** | **RD**  **(95% CI)** | **RR**  **(95% CI)** | **OR**  **(95% CI)** | **Direction.**  **p-value** |
| --- | --- | --- | --- | --- | --- | --- | --- |
| **Complications of kidney transplant** | PS-matched | 42.1% | 39.5% | +2.5%  (0.4, 4.7) | 1.06  (1.01–1.12) | 1.11  (1.02–1.21) | Favors KTA  P=0.022 |
|  | Pre-matching | 42.1% | 41.4% | +0.6%  (–1.0, 2.3) | 1.02  (0.98–1.06) | 1.03  (0.96–1.10) | Neutral.  p=0.439 |
| **Hospital readmission** | PS-matched | 43.3% | 40.9% | +2.4%  (0.2, 4.6) | 1.06  (1.01–1.11) | 1.1  (1.01–1.21) | Favors KTA.  p=0.03 |
|  | Pre-matching | 43.3% | 40.0% | +3.2%  (1.6, 4.9) | 1.08  (1.04–1.12) | 1.14  (1.07–1.22) | Favors KTA.  p<0.0001 |
| **MAKE** | PS-matched | 53.7% | 56% | –2.4%  (–4.6, -0.2) | 0.96  (0.92–1) | 0.91  (0.83–0.99) | Favors SPKT.  p=0.033 |
|  | Pre-matching | 53.7% | 62.3% | –8.7%  (–10.3, –7.0) | 0.86  (0.83–0.89) | 0.70  (0.65–0.75) | Favors SPKT.  p<0.0001 |
| **Treated Acute Rejection** | PS-matched | 22.0% | 20.8% | +1.2%  (–0.6, 3) | 1.05  (0.97–1.15) | 1.07  (0.97–1.19) | Neutral.  p=0.2 |
|  | Pre-matching | 22.0% | 17.8% | +4.1%  (2.8, 5.5) | 1.23  (1.16–1.31) | 1.30  (1.20–1.41) | Favors KTA.  p<0.0001 |
| **Hypoglycaemia** | PS-matched | 6.0% | 5.9% | +0.1%  (–1.0, 1.1) | 1.01  (0.85–1.21) | 1.01  (0.84–1.22) | Neutral:  p=0.887 |
|  | Pre-matching | 6.0% | 6.0% | –0.0%  (–0.8, 0.8) | 1.00  (0.88–1.14) | 1.00  (0.87–1.15) | Neutral  p=0.976 |
| **Infection or Sepsis** | PS-matched | 13.4% | 11.7% | +1.7%  (0.2, 3.1) | 1.15  (1.02–1.29) | 1.17  (1.02–1.33) | Favors KTA  p=0.022 |
|  | Pre-matching | 13.4% | 12.6% | +0.8%  (–0.3, 1.9) | 1.07  (0.98–1.16) | 1.08  (0.98–1.19) | Neutral  p=0.145 |
| **Ketoacidosis / hyperosmolarity** | PS-matched | 3.4% | 3.6% | –0.2%  (–1.0, 0.6) | 0.94  (0.75–1.18) | 0.94  (0.74–1.19) | Neutral  p=0.586 |
|  | Pre-matching | 3.4% | 3.2% | +0.3%  (–0.3, 0.9) | 1.08  (0.91–1.29) | 1.08  (0.90–1.30) | Neutral.  p=0.393 |

Abbreviations. SPKT, simultaneous pancreas–kidney transplant; KTA, kidney transplant alone; AR, absolute risk; RD, risk difference; RR, relative risk; OR odds ratio; CI, confidence interval; PS-matched, propensity-score matched; NS, not significant; MAKE, major adverse kidney events.

**Supplementary Table 3. Five-year absolute and relative risks (SPKT vs KTA)**

| **Outcome** | **Cohort** | **AR SPKT**  **(5y)** | **AR KTA**  **(5y)** | **RD**  **(95% CI)** | **RR**  **(95% CI)** | **OR**  **(95% CI)** | **Direction.**  **p-value** |
| --- | --- | --- | --- | --- | --- | --- | --- |
| **All-cause mortality** | PS-matched | 9.4% | 8.8% | +0.5%  (-0.7,1.8) | 1.06  (0.92, 1.22) | 1.07  (0.92, 1.24) | Neutral.  p=0.415 |
|  | Pre-matching | 9.4% | 11.6% | -2.2%  (-3.2, -1.3) | 0.81  (0.73, 0.89) | 0.79  (0.70, 0.88) | Favors SPKT.  p=0.000 |
| **Kidney graft failure** | PS-matched | 33.1% | 33.5% | -0.4%  (-2.5, 1.6) | 0.99  (0.93, 1.05) | 0.98  (0.89, 1.08) | Neutral.  p=0.335 |
|  | Pre-matching | 33.1% | 32.8% | +0.3%  (-1.3, 1.9) | 1.01  (0.98,1.04) | 1.01  (0.98,1.05) | Neutral.  p=0.714 |
| **Death-censored graft failure** | PS-matched | 28.1% | 28.3% | -0.2%  (-2.2. 1.7) | 0.99  (0.92, 1.06) | 0.99  (0.90, 1.09) | Neutral.  p=0.823 |
|  | Pre-matching | 28.1% | 25.1% | +3.0%  (1.5, 4.4) | 1.12  (1.06, 1.18) | 1.16  (1.08, 1.25) | Favors KTA.  p=0.000 |
| **Treated Acute Rejection** | PS-matched | 28.3% | 28.1% | +0.2%  (-1.7, 2.2) | 1.01  (0.94, 1.08) | 1.01  (0.92, 1.12) | Neutral.  p=0.804 |
|  | Pre-matching | 28.3% | 23.4% | +4.9%  (3.4, 6.3) | 1.21  (1.14, 1.27) | 1.29  (1.2, 1.39) | Favors KTA.  p=0.000 |
| **Complication of kidney**  **Transplant** | PS-matched | 52.5% | 49.7% | +2.7%  (0.6, 4.9) | 1.06  (1.01, 1.10) | 1.12  (1.02, 1.22) | Favors KTA.  p=0.014 |
|  | Pre-matching | 52.5% | 49.5% | +3.0%  (1.3, 4.6) | 1.06  (1.03, 1.09) | 1.13  (1.05, 1.20) | Favors KTA.  p=0.000 |
| **Depression/Anxiety**  **onset post-Tx** | PS-matched | 14.8% | 15.1% | -0.4%  (-2.1, 1.4) | 0.98  (0.87, 1.1) | 0.97  (0.85, 1.12) | Neutral.  p=0.683 |
|  | Pre-matching | 14.8% | 12.9% | +1.9%  (0.5, 3.2) | 1.15  (1.04, 1.25) | 1.17  (1.05, 1.30) | Favors KTA.  p=0.004 |
| **MAKE** | PS-matched | 63% | 64.5% | -1.5%  (-3.7, 0.6) | 0.98  (0.94, 1.01) | 0.94  (0.85, 1.02) | Neutral.  p=0.150 |
|  | Pre-matching | 63.0% | 68.3% | -5.3%  (-6.9, -3.8) | 0.92  (0.90, 0.95) | 0.79  (0.74, 0.85) | Favors SPKT.  P<0.0001 |
| **Post-transplant**  **cardiovascular events** | PS-matched | 27.6% | 28.2% | -0.5%  (-2.5, 1.4) | 0.98  (0.91, 1.05) | 0.97  (0.88–1.07) | Neutral.  p=0.584 |
|  | Pre-matching | 27.6% | 34.8% | -7.2%  (-8.7, -5.7) | 0.79  (0.75, 0.84) | 0.71  (0.66, 0.77) | Favors SPKT.  p=0.000 |
| **Acute Myocardial infarction**  **(first episode)** | PS-matched | 6% | 5.5% | +0.5%  (-0.5, 1.6) | 1.09  (0.91, 1.31) | 1.1  (0.90, 1.33) | Neutral.  p=0.344 |
|  | Pre-matching | 6% | 5.9% | -0.1%  (-0.8, 0.9) | 1.01  (0.88, 1.16) | 1.01  (0.88, 1.17) | Neutral.  p=0.887 |
| **Heart Failure**  **(first episode)** | PS-matched | 9.3% | 10.5% | -1.2%  (-2.6, 0.2) | 0.89  (0.77, 1.02) | 0.88  (0.75, 1.03) | Neutral.  p=0.099 |
|  | Pre-matching | 9.3% | 13.4% | -4.1%  (-5.2, -3.0) | 0.70  (0.62, 0.78) | 0.66  (0.59, 0.75) | Favors SPKT.  P<0.0001 |
| **Stroke**  **(first episode)** | PS-matched | 3.5% | 3.3% | +0.2%  (-0.6, 1.0) | 1.06  (0.84, 1.35) | 1.07  (0.83, 1.36) | Neutral.  p=0.611 |
|  | Pre-matching | 3.5% | 3.7% | -0.2%  (-0.8, 0.4) | 0.94  (0.79, 1.12) | 0.94  (0.78, 1.13) | Neutral.  p=0.498 |
| **Infection or Sepsis** | PS-matched | 20.9% | 20.6% | +0.3%  (-1.5, 2.1) | 1.015  (0.93, 1.11) | 1.02  (0.91, 1.14) | Neutral.  p=0.741 |
|  | Pre-matching | 20.9% | 22.0% | -1.1%  (-2.5, 0.2) | 0.95  (0.89, 1.01) | 0.94  (0.86, 1.02) | Neutral.  p=0.114 |
| **Hypoglycaemia** | PS-matched | 10.7% | 10.3% | +0.4%  (-0.9, 1.8) | 1.04  (0.92, 1.19) | 1.05  (0.91, 1.21) | Neutral.  p=0.512 |
|  | Pre-matching | 10.7% | 10.6% | +0.1%  (-0.9, 1.1) | 1.01  (0.92, 1.11) | 1.01  (0.91, 1.13) | Neutral.  p=0.837 |
| **Ketoacidosis-hyperosmolarity** | PS-matched | 7.5% | 7.8% | -0.2%  (-1.4, 0.9) | 0.97  (0.83, 1.13) | 0.97  (0.82, 1.14) | Neutral.  p=0.705 |
|  | Pre-matching | 7.6% | 5.8% | +1.8%  (0.9, 2.6) | 1.30  (1.16, 1.47) | 1.33  (1.17, 1.51) | Favors KTA.  p=0.000 |
| **Diabetic neuropathy**  **(new onset)** | PS-matched | 19.5% | 17.8% | +1.7%  (-0.5, 3.8) | 1.09  (0.98, 1.23) | 1.12  (0.97, 1.28) | Neutral.  p=0.128 |
|  | Pre-matching | 19.5% | 17.3% | +2.2%  (0.5, 3.8) | 1.13  (1.03, 1.23) | 1.16  (1.04, 1.29) | Favors KTA.  p=0.007 |
| **Diabetic retinopathy**  **(new onset)** | PS-matched | 14.5% | 13.5% | +1.1%  (-0.8, 2.9) | 1.08  (0.95, 1.23) | 1.09  (0.94, 1.27) | Neutral.  p=0.223 |
|  | Pre-matching | 14.5% | 12.3% | +2.2%  (0.8, 3.6) | 1.18  (1.07, 1.31) | 1.21  (1.08, 1.36) | Favors KTA.  p=0.012 |
| **PTLD or neoplasm**  **(new onset)** | PS-matched | 20.5% | 20.0% | +0.5%  (-1.4, 2.4) | 1.03  (0.93, 1.13) | 1.032  (0.92, 1.16) | Neutral.  p=0.612 |
|  | Pre-matching | 20.5% | 19.9% | +0.6%  (-0.9, 2.1) | 1.03  (0.96, 1.11) | 1.04  (0.95, 1.14) | Neutral.  p=0.416 |

Abbreviations. SPKT, simultaneous pancreas–kidney transplant; KTA, kidney transplant alone; KM, Kaplan–Meier; HR, hazard ratio; CI, confidence interval; PS-matched, propensity-score matched; MAKE, major adverse kidney events; PTLD, post-transplant lymphoproliferative disorder

**Supplementary Table 4. Ten-year absolute and relative risks (SPKT vs KTA)**

| **Outcome** | **Cohort** | **AR SPKT**  **(10y)** | **AR KTA**  **(10y)** | **RD**  **(95% CI)** | **RR**  **(95% CI)** | **OR**  **(95% CI)** | **Direction.**  **p-value** |
| --- | --- | --- | --- | --- | --- | --- | --- |
| **All-cause mortality** | PS-matched | 16.1% | 16.2% | -0.0% (-1.7, 1.6) | 1.00 (0.90, 1.10) | 1.00 (0.89, 1.12) | Neutral.  p=0.952 |
|  | Pre-matching | 16.1% | 18.4% | -2.3% (-3.5, -1.1) | 0.88 (0.81, 0.94) | 0.85 (0.78, 0.93) | Favors SPKT.  p<0.001 |
| **Kidney graft failure** | PS-matched | 39.9% | 40.4% | -0.4% (-2.6, 1.7) | 0.99 (0.94, 1.04) | 0.98 (0.90, 1.07) | Neutral.  p=0.682 |
|  | Pre-matching | 39.9% | 39.8% | +0.1% (-1.5, 1.8) | 1.00 (0.96, 1.04) | 1.01 (0.94, 1.08) | Neutral.  p=0.859 |
| **Death-censored graft failure** | PS-matched | 32.5% | 32.4% | +0.1% (-2.0, 2.1) | 1.00 (0.94, 1.07) | 1.00 (0.91, 1.10) | Neutral.  p=0.943 |
|  | Pre-matching | 32.5% | 28.9% | +3.6% (2.0, 5.1) | 1.12 (1.07, 1.18) | 1.18 (1.10, 1.27) | Favors KTA.  p<0.001 |
| **Treated Acute Rejection** | PS-matched | 31.5% | 31.0% | +0.5% (-1.5, 2.6) | 1.02 (0.95, 1.09) | 1.03 (0.93, 1.13) | Neutral.  p=0.596 |
|  | Pre-matching | 31.5% | 26.0% | +5.5% (4.4, 6.6) | 1.21 (1.16, 1.27) | 1.29 (1.21, 1.37) | Favors KTA.  p<0.001 |
| **Complication of kidney**  **Transplant** | PS-matched | 58.3% | 55.7% | +2.6% (0.5, 4.8) | 1.05 (1.01, 1.09) | 1.11 (1.02, 1.22) | Favors KTA.  p=0.018 |
|  | Pre-matching | 58.3% | 54.9% | +3.4% (1.8, 5.1) | 1.06 (1.03, 1.09) | 1.15 (1.07, 1.23) | Favors KTA.  p<0.001 |
| **Depression/Anxiety**  **onset post-Tx** | PS-matched | 19.7% | 19.8% | -0.1% (-2.1, 1.9) | 1.00 (0.90, 1.10) | 0.99 (0.88, 1.13) | Neutral.  p=0.932 |
|  | Pre-matching | 19.7% | 16.6% | +3.1% (1.6, 4.6) | 1.19 (1.10, 1.28) | 1.23 (1.12, 1.36) | Favors KTA.  p<0.001 |
| **MAKE** | PS-matched | 69.1% | 70.4% | -1.3% (-3.3, 0.7) | 0.98 (0.95, 1.01) | 0.94 (0.85, 1.03) | Neutral.  p=0.197 |
|  | Pre-matching | 69.1% | 72.9% | -3.9% (-5.4, -2.3) | 0.95 (0.93, 0.97) | 0.83 (0.77, 0.89) | Favors SPKT.  p<0.001 |
| **Post-transplant**  **cardiovascular events** | PS-matched | 35.0% | 35.3% | -0.2% (-2.3, 1.8) | 0.99 (0.94, 1.05) | 0.99 (0.90, 1.08) | Neutral.  p=0.815 |
|  | Pre-matching | 35.0% | 41.2% | -6.2% (-7.8, -4.6) | 0.85 (0.81, 0.89) | 0.77 (0.72, 0.82) | Favors SPKT.  p<0.001 |
| **Acute Myocardial infarction**  **(first episode)** | PS-matched | 9.2% | 8.4% | +0.8% (-0.4, 2.1) | 1.07 (0.97, 1.18) | 1.08 (0.96, 1.21) | Neutral.  p=0.204 |
|  | Pre-matching | 9.2% | 8.9% | +0.3% (-0.8, 1.4) | 1.03 (0.94, 1.14) | 1.04 (0.94, 1.15) | Neutral.  p=0.565 |
| **Heart Failure**  **(first episode)** | PS-matched | 15.1% | 15.6% | -0.5% (-2.2, 1.2) | 0.97 (0.87, 1.08) | 0.96 (0.85, 1.10) | Neutral.  p=0.598 |
|  | Pre-matching | 15.1% | 19.1% | -3.9% (-5.2, -2.6) | 0.79 (0.73, 0.86) | 0.76 (0.69, 0.84) | Favors SPKT.  p<0.001 |
| **Stroke**  **(first episode)** | PS-matched | 5.7% | 5.3% | +0.5% (-0.5, 1.5) | 1.09 (0.91, 1.31) | 1.09 (0.90, 1.33) | Neutral.  p=0.363 |
|  | Pre-matching | 5.7% | 5.6% | +0.2% (-0.6, 1.0) | 1.03 (0.90, 1.18) | 1.03 (0.89, 1.19) | Neutral.  p=0.680 |
| **Infection or Sepsis** | PS-matched | 27.3% | 27.2% | +0.1% (-1.9, 2.0) | 1.00 (0.93, 1.08) | 1.00 (0.91, 1.11) | Neutral.  p=0.940 |
|  | Pre-matching | 27.3% | 27.8% | -0.5% (-2.0, 1.0) | 0.98 (0.93, 1.04) | 0.98 (0.91, 1.05) | Neutral.  p=0.519 |
| **Hypoglycaemia** | PS-matched | 13.8% | 13.4% | +0.3% (-1.2, 1.8) | 1.02 (0.92, 1.14) | 1.03 (0.91, 1.17) | Neutral.  p=0.672 |
|  | Pre-matching | 13.8% | 13.5% | +0.3% (-0.9, 1.4) | 1.02 (0.94, 1.11) | 1.02 (0.93, 1.13) | Neutral.  p=0.652 |
| **Ketoacidosis-hyperosmolarity** | PS-matched | 9.5% | 9.9% | -0.4% (-1.7, 0.9) | 0.96 (0.84, 1.09) | 0.95 (0.82, 1.10) | Neutral.  p=0.521 |
|  | Pre-matching | 9.5% | 7.2% | +2.3% (1.3, 3.3) | 1.32 (1.19, 1.47) | 1.35 (1.21, 1.52) | Favors KTA.  p= p<0.001 |
| **Diabetic neuropathy**  **(new onset)** | PS-matched | 24.3% | 22.4% | +1.9% (-0.4, 4.2) | 1.08 (0.98, 1.20) | 1.11 (0.98, 1.27) | Neutral.  p=0.107 |
|  | Pre-matching | 24.3% | 21.8% | +2.5% (0.7, 4.3) | 1.11 (1.03, 1.20) | 1.15 (1.04, 1.27) | Favors KTA.  p=0.005 |
| **Diabetic retinopathy**  **(new onset)** | PS-matched | 17.5% | 16.7% | +0.8% (-1.2, 2.8) | 1.05 (0.93, 1.18) | 1.06 (0.92, 1.22) | Neutral.  p=0.414 |
|  | Pre-matching | 17.5% | 15.2% | +2.4% (0.8, 3.9) | 1.16 (1.06, 1.26) | 1.19 (1.07, 1.32) | Favors KTA.  p=0.002 |
| **PTLD or neoplasm**  **(new onset)** | PS-matched | 26.5% | 26.1% | +0.4% (-1.7, 2.5) | 1.01 (0.94, 1.10) | 1.02 (0.91, 1.14) | Neutral.  p=0.721 |
|  | Pre-matching | 26.5% | 25.3% | +1.2% (-0.4, 2.8) | 1.05 (0.98, 1.12) | 1.06 (0.97, 1.15) | Neutral.  p=0.140 |

Abbreviations: SPKT, simultaneous pancreas–kidney transplant; KTA, kidney transplant alone; AR, absolute risk; RD, risk difference; RR, relative risk; OR, odds ratio; CI, confidence interval; PS-matched, propensity-score matched; MAKE, major adverse kidney events; PTLD, post-transplant lymphoproliferative disorder. Risk difference is expressed as AR SPKT – AR KTA. Direction indicates the favoured strategy (SPKT or KTA) or neutrality based on statistical significance.

**Supplementary table 5. Longitudinal outcomes (Kaplan–Meier and Cox models): SPKT (n=2,760; median follow-up 5.8 yrs) vs KTA (n=7,447; median follow-up 5.3 yrs) in T1D**

| Outcome | Cohort | Hazard Ratio  (95% CI) | KM  log-rank p | Direction |
| --- | --- | --- | --- | --- |
| All-cause mortality | PS-matched | 0.95 (0.84–1.06) | 0.36 | Neutral |
|  | Pre-matching | 0.80 (0.73–0.88) | <0.001 | Favors PKT |
| Kidney graft failure | PS-matched | 0.97 (0.89–1.05) | 0.41 | Neutral |
|  | Pre-matching | 0.91 (0.87–0.97) | 0.01 | Favors SPKT |
| Death-censored graft failure | PS-matched | 0.99 (0.91–1.09) | 0.87 | Neutral |
|  | Pre-matching | 1.00 (0.93–1.08) | 0.95 | Neutral |
| MAKE | PS-matched | 0.96 (0.90–1.02) | 0.20 | Neutral |
|  | Pre-matching | 0.86 (0.82–0.91) | <0.001 | Favors SPKT |
| Post-transplant  cardiovascular events | PS-matched | 1.00 (0.92–1.09) | 0.96 | Neutral |
|  | Pre-matching | 0.75 (0.70–0.80) | <0.001 | Favors SPKT |
| Treated acute rejection | PS-matched | 1.04 (0.95–1.14) | 0.41 | Neutral |
|  | Pre-matching | 1.10 (1.02–1.19) | 0.02 | Favors KTA |
| Acute myocardial infarction (first event) | PS-matched | 0.96 (0.81–1.14) | 0.64 | Neutral |
|  | Pre-matching | 0.77 (0.67–0.89) | <0.001 | Favors SPKT |
| Heart failure (first event) | PS-matched | 0.96 (0.84–1.10) | 0.56 | Neutral |
|  | Pre-matching | 0.76 (0.68–0.85) | <0.001 | Favors SPKT |

Abbreviations. SPKT, simultaneous pancreas–kidney transplant; KTA, kidney transplant alone; KM, Kaplan–Meier; HR, hazard ratio; CI, confidence interval; PS-matched, propensity-score matched; MAKE, major adverse kidney events; PTLD, post-transplant lymphoproliferative disorder

**Supplementary table 6. Longitudinal outcomes (Kaplan–Meier and Cox models): SPKT (n=1,456; median follow-up 5.7 yrs) vs KTA (n=3,365; median follow-up 5.2 yrs) in T1D with BMI ≤30**

| Outcome | Cohort | Hazard Ratio  (95% CI) | KM  log-rank p | Direction |
| --- | --- | --- | --- | --- |
| All-cause mortality | PS-matched | 1.003 (0.855–1.177) | 0.9711 | Neutral |
|  | Pre-matching | 0.805 (0.704–0.919) | 0.0013 | Favors SPKT |
| Kidney graft failure | PS-matched | 0.973 (0.872–1.085) | 0.6218 | Neutral |
|  | Pre-matching | 0.879 (0.802–0.964) | 0.0060 | Favors SPKT |
| Death-censored graft failure | PS-matched | 0.993 (0.908–1.085) | 0.8705 | Neutral |
|  | Pre-matching | 0.973 (0.859–1.101) | 0.6605 | Neutral |
| MAKE | PS-matched | 0.958 (0.897–1.043) | 0.3247 | Neutral |
|  | Pre-matching | 0.874 (0.813–0.94) | 0.0002 | Favors SPKT |
| Post-transplant  cardiovascular events | PS-matched | 1.008 (0.894–1.136) | 0.9012 | Neutral |
|  | Pre-matching | 0.764(0.692–0.843) | <0.0001 | Favors SPKT |
| Treated acute rejection | PS-matched | 0.973 (0.855–1.107) | 0.6730 | Neutral |
|  | Pre-matching | 1.046 (0.936–1.169) | 0.4306 | Neutral |
| Acute myocardial infarction (first event) | PS-matched | 0.993 (0.774–1.274) | 0.9535 | Neutral |
|  | Pre-matching | 0.747 (0.608–0.918) | 0.0053 | Favors SPKT |
| Heart failure (first event) | PS-matched | 1.002 (0.825–1.216) | 0.9868 | Neutral |
|  | Pre-matching | 0.758 (0.645–0.89) | 0.0007 | Favors SPKT |

Abbreviations. SPKT, simultaneous pancreas–kidney transplant; KTA, kidney transplant alone; KM, Kaplan–Meier; HR, hazard ratio; CI, confidence interval; PS-matched, propensity-score matched; MAKE, major adverse kidney events; PTLD, post-transplant lymphoproliferative disorder
